# Context-Aware Automatic Sign Language Video Transcription in Psychiatric Interviews †

**DOI:** 10.3390/s22072656

**Published:** 2022-03-30

**Authors:** Erion-Vasilis Pikoulis, Aristeidis Bifis, Maria Trigka, Constantinos Constantinopoulos, Dimitrios Kosmopoulos

**Affiliations:** Computer Engineering and Informatics Department, University of Patras, 26504 Patras, Greece; pikoulis@ceid.upatras.gr (E.-V.P.); bifis@ceid.upatras.gr (A.B.); kkonstantino@upatras.gr (C.C.); dkosmo@upatras.gr (D.K.)

**Keywords:** sign language recognition, sign language datasets, machine learning

## Abstract

Sign language (SL) translation constitutes an extremely challenging task when undertaken in a general unconstrained setup, especially in the absence of vast training datasets that enable the use of end-to-end solutions employing deep architectures. In such cases, the ability to incorporate prior information can yield a significant improvement in the translation results by greatly restricting the search space of the potential solutions. In this work, we treat the translation problem in the limited confines of psychiatric interviews involving doctor-patient diagnostic sessions for deaf and hard of hearing patients with mental health problems.To overcome the lack of extensive training data and be able to improve the obtained translation performance, we follow a domain-specific approach combining data-driven feature extraction with the incorporation of prior information drawn from the available domain knowledge. This knowledge enables us to model the context of the interviews by using an appropriately defined hierarchical ontology for the contained dialogue, allowing for the classification of the current state of the interview, based on the doctor’s question. Utilizing this information, video transcription is treated as a sentence retrieval problem. The goal is predicting the patient’s sentence that has been signed in the SL video based on the available pool of possible responses, given the context of the current exchange. Our experimental evaluation using simulated scenarios of psychiatric interviews demonstrate the significant gains of incorporating context awareness in the system’s decisions.

## 1. Introduction

The Deaf (with a capital D) are defined as a group of people with varying hearing acuity, whose primary mode of communication is a visual language, predominantly sign language (SL), and who have a shared heritage and culture. There are 70 million deaf and hard of hearing people worldwide, and more than 200 officially recognized national sign languages [1]. Unfortunately, most Deaf are not able to use their native SLs in their interactions with the non-Deaf, instead being limited to other communication methods such as writing or texting. However, most Deaf prefer to express themselves in their native SLs and often avoid using writing/reading due to their rather poor written language skills [2]. The situation is worsened by the scarcity of dedicated SL interpreters who could help alleviate the issue, especially in critical situations (e.g., health services, court, etc.) via their live presence or through relay services. For example, it is estimated that in the European Union, there are only 12,000 registered interpreters serving more than 750,000 deaf SL users [3]. This is indicative of the many communication barriers that exist for deaf SL users.

To help mitigate the problem, automated translation systems are gaining both in popularity and in performance, especially since the advent and widespread use of deep neural networks. However, despite the progress, automatic SL translation (SLT) remains an open and extremely challenging task, particularly when attempted under a general unconstrained framework, whose treatment requires an interdisciplinary approach involving linguistics for identifying the structures of SL; natural language processing (NLP) and machine translation (MT) for modeling, analyzing, and translating; and computer vision for detecting signed content [4].

It must be stressed that even at the level of individual sign recognition, SL translation presents itself with a number of difficulties due to the fact that each sign is expressed via a multitude of information streams involving hand shapes and facial expressions (including eyebrows, mouth, head, and eye gaze) as well as secondary streams such as, e.g., the movement of shoulders [5]. Adding to the problem is the extensive use of depiction, namely using the body to depict actions, dialogues, or psychological events [6], which occurs very frequently in SL. Taking into account that (in direct analogy to spoken languages) a real-world translation system requires continuous SL recognition (i.e., translating a continuous stream of signs) [7], which is further complicated by epenthetic effects (insertion of extra features into signs), coarticulation (the ending of one sign affecting the start of the next), and spontaneous sign production (which may include slang, nonuniform speed, etc.), the sheer magnitude of the problem becomes readily apparent [8].

Recent methods based on networks with self-attention (transformers) [9,10], which currently represent the state-of-the-art in SLT, have yielded promising results owing to their ability to learn without depending too much on expert knowledge. Nevertheless, to fully unleash their performance and generalization potential, such systems require large corpora for training, which increase with the size of the vocabulary. This is a well-known issue faced by all data-driven approaches based on deep learning regardless of application. However, contrary to other domains such as speech processing that are endowed with almost unlimited training data, the issue becomes especially critical in the context of SLT, where there is a profound lack of annotated data for supervised training because of the very complicated language structures that SLs entail and also because almost all SLs are minority languages. As a result, the currently available SL benchmarks such as the PHOENIX-2014 [11] and the SIGNUM [12] datasets are several orders of magnitude smaller than similarly defined speech-related corpora [4], which drastically restricts the generalization capability of models for unseen situations/signers.

In this work, we maintain that the complexity of the translation task, combined with data scarcity, necessitates encoding and utilizing all a priori available knowledge, given that generating large annotated SL datasets can be extremely time-consuming and expensive. This prior information includes linguistic structures and/or domains and context knowledge and can be incorporated in the form of constraints that guide the solution by effectively limiting the required search space.The highlights of this paper can be summarized as follows:

- We present an SL translation framework aimed at enhancing the mental health services provided to deaf or hard of hearing people by facilitating the communication between health professionals and deaf patients suffering from anxiety disorders, stress, and depression.
- We propose a domain-specific solution combining data-driven feature extraction (using the deep-learning-based MediaPipe [13] tool) with the encoding and utilization of a priori information stemming from the available domain knowledge to combat the lack of extensive training datasets.
- The knowledge regarding the vocabulary used, as well as the flow and structure of information (which is dictated by the format of a doctor-patient dialogue), enables us to define a suitable hierarchical ontology (first proposed in our previous work [14], and presented here in Section 4). We can then combine this ontology with a set of classification approaches in order to model the context of the exchange by labeling the the dialogue acts that take place during the interview.
- The translation task itself is treated as a sentence retrieval problem whereby the problem is reduced to identifying the known response that best matches the unknown one given the context of the dialogue.
- Our experiments are conducted using an in-house-created dataset consisting of 21 simulated psychiatric interviews, each of them signed by a combination of (8) native and experienced users of the Greek sign language (GSL).

This paper is structured as follows. In Section 2, we present some of the most important recent works in the field of SL translation/recognition. In Section 3, we introduce the framework of the psychiatric interviews used in this paper. In Section 4 and Section 5, we present in detail the proposed techniques for context modeling and context-aware sentence recognition, respectively, while our experimental results are presented in Section 6. In Section 7, we hold a brief discussion regarding the highlights and shortcomings of the proposed work, and finally, Section 8 contains our conclusions.

## 2. Related Work

Sign language translation has been commonly regarded as a recognition problem (see [15,16] for details). Early approaches attempted to recognize individual and well-segmented signs by employing discriminative or generative methods within a time-series classification framework; examples include hidden Markov models (HMMs), e.g., [17,18,19], dynamic time warping, e.g., [20,21], and conditional random fields, e.g., [22,23]. These methods used handcrafted features; more recently, deep learning methods such as those derived from CNNs, provided some superior representations, e.g., [24,25].

The recognition approach, however, has rather limited real-world utility because it produces a group of words with relatively nonsensical context structure rather than a natural language output. As a result, SLT with continuous recognition is a much more realistic framework, but it is also far more difficult to implement [8,26,27]. The difficulty stems from epenthesis (the incorporation of extra visual clues into signs), coarticulation (the conclusion of one sign affects the beginning of the next), and spontaneous sign generation (which may include slang, special expressions, etc.). In [28], the authors used a model comprised of a CNN-LSTM network to produce features, which were then fed to HMMs that provided inference using a variation of the Viterbi method to handle the challenge. A 2D-CNN with cascaded 1D convolutional layers for feature extraction was proposed in [29], also using a bidirectional LSTM (BLSTM) for continuous SL recognition, and utilizing the Levenshtein distance to produce gloss-level alignments. Along the same lines, the authors in [30], combined a 2D fully convolutional network with a feature enhancement module to obtain better gloss alignments. In [31], the authors employed a BLSTM fed with CNN features, while [32] utilized an adaptive encoder-decoder architecture leveraging a hierarchical BLSTM with attention over sliding windows on the decoder. A network called STMC was proposed in [33], which incorporated several cues from position and picture (hands, face, holistic) in multiple scales and fed them to a CTC penultimate layer.

The recently proposed Transformer architectures enable SLT to drastically enhance translation performance. This is amplified when SLT is combined with an SLR procedure, either as an intermediate activity or in the context of a multitask learning scheme. In particular, in [9], the authors used a Transformer network to achieve end-to-end translation. They essentially suggested an S2(G+T) architecture. They proposed a Transformer network to conduct S2T, and they used the Transformer’s encoder to forecast the respective gloss sequence ground-truth. The latter SLR task was carried out over all potential gloss alignments by a penultimate connectionist temporal classification (CTC) layer [34]. Training was performed collaboratively for the entire system (both tasks). The need for that intermediate step has been alleviated in later works such as [10], where a winner-takes-all activation is integrated into the Transformer architecture. In [35], the authors introduced a context-aware continuous sign language recognition using a generative adversarial network architecture. The elaborated system exploits text or contextual information to enhance the recognition accuracy, in contrast to previous works that only considered spatio-temporal features from video sequences. In particular, it recognizes sign language glosses by extracting these features and assessing the prediction quality by modeling text information at the sentence and gloss levels.

Despite the aforementioned developments, such works still face issues in more complex real-world scenarios, mainly due to the lack of available data. They are most often implemented on small dictionaries relevant to certain real-world contexts for which very labor-intensive annotation has taken place, e.g., weather reports [11]. The question is how to use these advancements in real scenarios when not enough training data is available, but the structure of the conversation is more or less known, e.g., by following a protocol that can be modeled to a certain extent a priori. To our knowledge, there has been no such effort in the related literature for the SLT. This work aspires to contribute toward bridging this gap.

## 3. The Case of Psychiatric Interviews

Anxiety disorders, stress, and depression are quite common in the general population. They are associated with the modern way of life and often cause significant reduction in the individual’s functionality, resulting in notable burdens on health systems. Due to the close relationship and coexistence of anxiety and depressive disorders with physical ailments (either as a cause or as a consequence), the individual’s ability to access mental health services and the provision of appropriate psychiatric treatment are crucial factors in the control and prognosis of anxiety and depressive disorders. A prerequisite for the proper treatment of each individual is the collection of a detailed patient record through a psychiatric interview, which leads to appropriate diagnosis and treatment [36,37,38].

The test case that we examine in this paper regards psychiatric interviews, with the goal of developing a service that can yield real-time interpretations and facilitate doctor-patient communication. It has been selected due to its high impact and due to the structured approach that is commonly followed by doctors.

To achieve this goal, we modeled the context of the doctor-patient dialogues based on a hierarchy of dialogue acts (DA) [39], and we predict the expected vocabulary of the patient’s response to optimize the SL-to-text translation process. The task of SL-to-text translation is very challenging and typically requires computations over large vocabularies. Our approach aims to increase the quality of the translation by assigning greater probabilities to certain vocabulary terms, given the current context of the interview.

The dataset used for training/testing purposes consisted of simulated scenarios representing realistic interactions between mental health professionals and deaf patients suffering from anxiety disorders, stress, and depression. The scenarios were developed with the help of two professional psychiatrists. However, no actual human subjects (patients) were involved in creating the dataset. More specifically, the corpus contains 21 recorded scripts in the Greek language (GL), each of them signed by 8 users of Greek Sign Language (GSL). Of the users, 6 were native signers while 2 were experienced interpreters. It includes 1029 simple sentences with 945 of them being unique (excluding repetitions). Furthermore, the GL vocabulary includes 1374 unique words, while the total number of words is 6319. Hence, the average length of a sentence is 6.1 words. The words form 3558 unique 2-grams, 3841 unique 3-grams, and 3292 unique 4-grams. Moreover, the GSL vocabulary contains 806 unique glosses, while the complete corpus contains 2619 glosses. The average length of a sentence is 3.9 glosses. Finally, the glosses form 1666 unique 2-grams, 1337 unique 3-grams, and 870 unique 4-grams. Further details on the dataset can be found in [40].

## 4. Hierarchical Classification of Doctor–Patient Dialogue Acts

**Figure 1:**
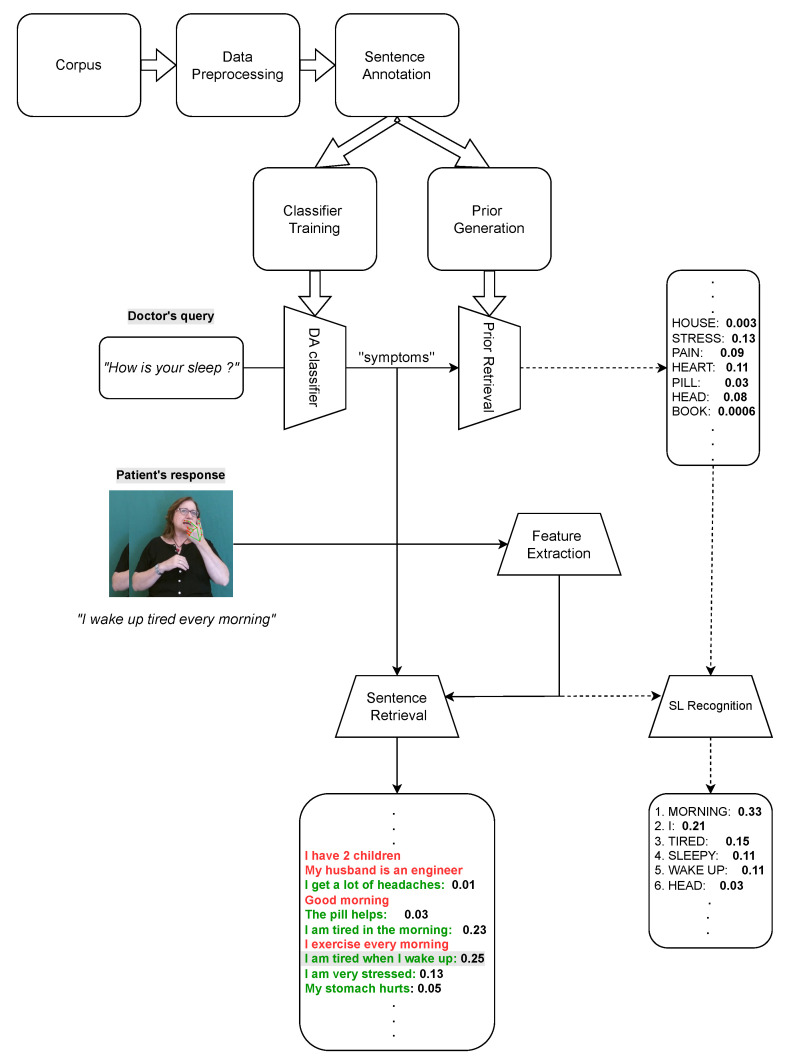
Architectural overview of the proposed framework. The dashed arrows represent the parts of the system that are currently under development.

In this section, we present a technique for doctor–patient dialogue modeling using a corpus of realistic scenarios for psychiatric interviews. To this end, we define a suitable ontology and propose a hierarchical classification scheme that accepts as input the doctor’s query and predicts the class to which the query belongs. Our aim is to create a complete system such as the one depicted in Figure 1. The system consists of several submodules, including a classifier to predict the topics of discussion and in turn select the appropriate prior for the expected vocabulary, and an SL-to-text translation network that utilizes this prior. When used in the context of a psychiatric session, the system can take the doctor’s query as input (using a speech-to-text tool) and feed it to the trained classifier to produce the predicted label/topic of discussion. This prediction is used to select the appropriate prior for the vocabulary terms to be translated. The patient response to the doctor, in the form of a SL video segment, is then given as input to the SL-to-text system, which incorporates the prior information to produce the translation. To train our classifiers and generate the term priors for each label/topic, we utilized the available dataset of realistic psychiatric interviews. The contained sentences were first preprocessed and then annotated using the topics defined in the proposed ontology. The latter takes the form of a directed acyclic graph (DAG) with the purpose of modeling the hierarchy of the topics typically found in a psychiatric interview. Finally the vocabulary of each label topic was formed based on the annotation, which was used as a way to generate the vocabulary term priors.

### 4.1. Dialogue Context

**Figure 2:**
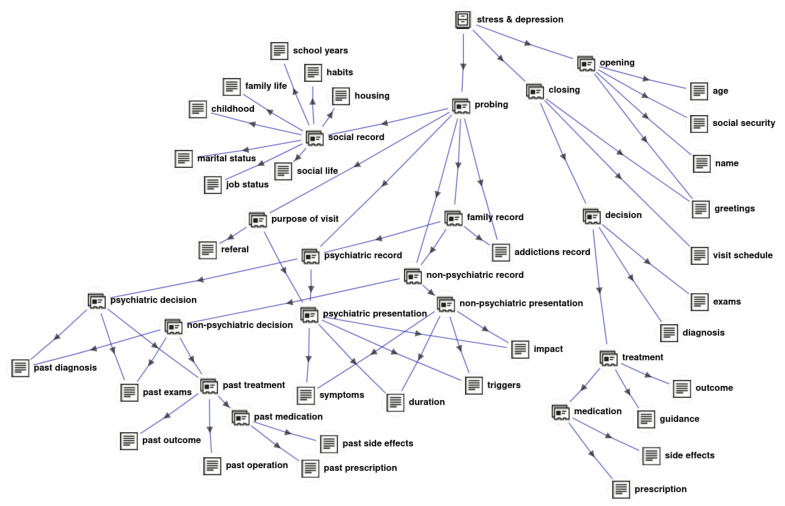
The proposed hierarchical ontology for labeling the parts of a psychiatric interview. Reprinted with permission from [14]. Copyright 2021 Association for Computing Machinery (ACM).

The task of assigning context to the parts of a dialogue is known as dialogue acts (DA) classification, e.g., [41,42,43]. The dialogue acts are essentially labels that characterize the type of exchange that is taking place, e.g., asking, refusing, giving directives, etc. More specifically, assuming a set $C=\{C_{1},C_{2},\cdots ,C_{N}\}$ of *N* dialogues, each consisting of a sequence of utterances (namely, sentences), i.e., $C_{i}=\left\{u_{1},u_{2},\cdots ,u_{N_{i}}\right\}$, and a set of *M* dialogue acts (labels) $Y=\{y_{1},y_{2},\cdots ,y_{M}\}$, the goal of the DA classification is to assign a label from *Y* to each utterance in C. Since we are interested in describing the context of an interview in more detail than the typical cases found in the literature, employing a set of generic DAs that could be used for everyday dialogues would not suffice for our purpose. To this end, after careful examination of the available collection of interviews, we propose the hierarchical ontology depicted in Figure 2 for the DAs found in our corpus.

The proposed ontology comes in the form of a directed acyclic graph (DAG), with stress & depression at its root, while the children DAs correspond to the main sections of each interview, namely opening, probing, and closing. Probing is in turn branching out to: purpose of visit, psychiatric record, nonpsychiatric record, social life record, family record, and so on. The fully expanded graph has 30 terminal nodes that correspond to the most detailed DAs (see Figure 2). The proposed ontology is the result of careful analysis on the available dataset, consisting of realistic doctor–patient dialogue sessions. The leaves of the ontology represent the relevant topics that are discussed, typically found in such sessions guided by the psychiatrist. The structure depicted in Figure 2 showcases the interconnection between the topics within the context of the domain. Every node in the graph represents a subset of topics that form the node’s parents. We consider the root node “stress and depression” as the superset and the leaf labels as singletons. The ontology’s primary purpose is to assist the hierarchical classification process which is presented in the following sections in detail.

Equipped with the DA graph, we assigned one leaf label to each question of the psychiatrist in our corpus. Noting that a psychiatric interview has a rather strict structure guided by the questions of the psychiatrist, we avoided explicitly classifying the patient’s responses and assumed that the DA of the patient’s response is determined by the preceding question. An example of DA classification containing an annotated excerpt from our corpus, is shown in Table 1.

In the following subsection, we present the proposed methodology for classifying new questions. It consists of three main stages, namely, data preprocessing, feature extraction, and classification.

### 4.2. Data Preprocessing

The main preprocessing steps on the available interviews are the following. First, we organized the sentences (namely, the utterances $u_{i}$) of all scripts into two types of DAs, i.e., doctor queries and patient responses. All sentences were originally recorded in Greek and then translated to English using machine translation software.

Then, we annotated all sentences (both the queries and the responses) with a label that best describes the context of the corresponding DA according to the ontology labels shown in Figure 2. By annotating all the query–response DAs, several groups of sentences for each DA were derived. Such knowledge gives us an insight on the per-class vocabulary prior and will be exploited in the SL-to-text translation process.

### 4.3. Sentence Embeddings

Following the preprocessing step, the doctor’s sentences were suitably transformed to facilitate our classification goal utilizing the representation power of deep neural networks. To this end, we employed Sentence-BERT (SBERT) [44], namely a modification of the pretrained BERT network [45] that uses siamese and triplet network structures to derive semantically meaningful sentence embeddings. Specifically, we used the stsb-bert-base model from the SentenceTransformers framework based on PyTorch and Transformers to translate each varying-length sentence into a vector representation of size 768.

### 4.4. Classification

The classification module associates an unknown query to one or more members from a predefined set of classes according to its attributes. We experimented with two classification schemes, one hierarchical and one flat. In both cases, the classification was based on the 768-dimensional SBERT feature vector representation of the query.

In the flat classification scheme, there is no utilization of the interview structure. In this approach, every query is classified to the appropriate label based on the distance between its SBERT representation and the representations of each class member. Due to the limited number of samples for training, we resorted to a modified version of a *k*-Nearest Neighbors classifier to perform this classification.

The hierarchical classification scheme [46] exploits the relationships among the classes and organizes them into a hierarchy of levels according to the DAG structure of the proposed ontology shown in Figure 2 (for further details the reader is referred to [14]):

We trained one classifier per class, following a top-down approach, where a given decision leads us down a different classification path. To better understand how hierarchical classification operates, it is necessary to think of a hierarchical classifier as a tree. Every node of the tree (except for the leaves) is a standalone classifier that classifies a query to one of its child nodes. Thus, to train each node-classifier, we need to split the training data into subsets based on the node’s children. To this end, from all the training data, we select the set that contains all the sentences belonging to class labels (leaves) that are reachable from the particular node-classifier. Then, we further partition this set into subsets (one per each of the node’s children), each containing the sentences that belong to leaves that are reachable from a particular child of the node. Doing this for all the nodes, results in a system that can hierarchically classify a query. Each query starts at the root and follows a classification trail on our tree all the way down to a leaf.

The classification process of a new sentence query is summarized in Algorithm 1, whereby two core functions can be discerned. The first one, namely mean_distance(), takes as input the SBERT vector representations of the node sentences and the new query. It then calculates the mean euclidean distance between the query vector and the vectors of each of the node’s children. On the other hand, child_with_min_distance_from(), accepts a dictionary with the names of the children nodes as keys and the mean distances as values. It returns the key (child name to be used as index) with the minimum distance value.
**Algorithm 1** Hierarchical Classification**  procedure**Classify(query)      index←root_node      **while** indexnotleaf **do**          distances←{}          **for** child←children(index) **do**             sentences←sentences_of(child)             distances(child)←mean_distance(sentences,query)          **end for**          index←child_with_min_distance_from(distances)      **end while**      label←index      **return** label  **end procedure**

Although a node may be reached from different paths, the goal of the classifier is to output the correct class label, irrespective of the path followed, in the specific problem. During the interview process, when a new doctor query occurs, it is passed through the trained hierarchical classifier to produce the appropriate label. This information is vital since, due to the nature of the interviews, we can make a strong assumption that the patient response will belong to the same class as the one assigned to the doctor query. By predicting the response’s label, we have a straightforward solution to acquire prior knowledge that will be used later on in the SL translation process.

## 5. Context-Aware SL Sentence Recognition

In this section, we present an SL-sentence recognition system on the HealthSign dataset using the dialogue act classification tool described previously. The general idea behind the proposed system is to infer the dialogue–act class of the doctor’s query using the classification scheme presented in Section 4, and then utilize this prior knowledge to facilitate the automatic recognition of the patient’s response. Despite its simplicity, the presented system is capable of achieving promising accuracy levels. This can showcase the importance of incorporating prior knowledge toward facilitating the solution of extremely complicated problems such as the automatic interpretation of SL videos.

Due to the rather limited size of the dataset, we pursued a nondeep treatment of the problem, involving feature extraction and statistical modeling. Specifically, we first extracted hand-related features from each video frame, and subsequently we clustered the feature vectors to translate the sentence video into a sequence of latent hand-shapes. Finally, we eliminated the time parameter, borrowing concepts from document-processing techniques. The recognition task was performed by simply assigning the unknown test sentence to its closest neighbor from the (known) sentences in the training corpus. In the subsequent subsections, we describe each of the involved steps in detail.

### 5.1. Feature Extraction

The first processing step in the proposed pipeline involved passing the SL videos though the “Hands” module of Mepiapipe ([13,47]) to infer hand-landmark locations. Specifically, the MediaPipe Hands tool estimated 21 3D landmarks per hand for each video frame. Using these hand landmarks, we calculated the (15) fingertip distances corresponding to the signer’s dominant hand, while subsequently, the resulting distance vector was normalized. Thus, by following this procedure, we extracted a rotation-, translation-, and scale-invariant hand-related feature vector for each video frame of the available SL videos.

### 5.2. Sentence Modeling

As a dimensionality reduction step, we used our training data to estimate latent hand shapes by grouping the $N_{\mathrm{frames}}$ feature vectors of the dataset into a small number of *k* clusters with $k\ll N_{\mathrm{frames}}$. We anticipated the cluster centroids to represent the fundamental hand shapes that were present in our collection of SL videos (allowing also for transitional frames).

To model our SL sentence videos, we assigned each video frame to the cluster to which its corresponding feature vector belonged, thus transforming SL videos into a sequence of latent hand shapes (namely, cluster labels). In other words, the *i*-th sentence of the dataset, $i=1,\cdots ,N_{\mathrm{sentences}}$, was translated into a sequence $l_{1},l_{2},\cdots ,l_{N_{\mathrm{frames}}^{i}}$, $l_{j}\in \left\{1,\cdots ,k\right\}$, where $N_{\mathrm{frames}}^{i}$ is the number of frames in the *i*-th sentence’s video. In order to assess the similarity between SL sentences, we must take into account that SL sentence videos of the same sentence may have different lengths, and even more importantly, that the same sentence may be signed in many different ways (e.g., by altering the order of the contained glosses). To overcome this obstacle, we modeled each sentence via the histogram of the latent hand shapes that are present in it. To be more specific, sentence $s_{i}$ was modeled via the *k*-dimensional vector $h_{i}$, defined as:

(1) $$h_{i}\equiv {\left[f_{1}^{i},f_{2}^{i},\cdots ,f_{k}^{i}\right]}^{T},$$

where

(2) $$f_{j}^{i}=\frac{n_{j}^{i}}{N_{\mathrm{frames}}^{i}},$$

with $n_{j}^{i}$ denoting the number of appearances of the *j*-th latent hand shape in the SL video of the *i*-th sentence. Note that in the context of information retrieval, the histogram defined in (1) corresponds to a bag-of-words [48] model, with the sentences representing “documents” and the latent handshapes, the “words” that comprise them. We are going to use this analogy again in the subsequent section, where we define the distance metrics used in our experiments for sentence comparison.

### 5.3. Quantifying Sentence Similarity

Using the bag-of-words sentence modeling, we can define a simple sentence recognition system that assigns the unknown test sentence $\tilde{s}$ to its closest neighbor $s_{i^{*}}$ in the training dataset. In this subsection, we define the various distance metrics used for quantifying the similarity between sentence pairs.

#### 5.3.1. Sentence Distance Using Residual Norm

Considering the hand shape histograms of each SL sentence as vectors in *k*-dimensional space (where *k* denotes the number of clusters), we can define distance metrics between the test sentence $\tilde{s}$ and the *i*-th sentence in the training dataset, in the form of $L^{p}$-norms of the residual $\tilde{h}-h_{i}$ between the corresponding histograms, i.e.,:

(3) $$D_{p}\left(\tilde{s},s_{i}\right)\equiv \left|\right|\tilde{h}-h_{i}{\left|\right|}_{p}.$$

In our experiments we examined the $L^{1}$, $L^{2}$, and $L^{\infty }$ norms for our classification task, reflecting the mean, mean squared, and maximum values, respectively, of the absolute differences $\left|{\tilde{f}}_{l}-f_{l}^{i}\right|$, l=1,⋯,k, where ${\tilde{f}}_{l}$, $f_{l}^{i}$ denote the frequency of the *l*-th hand shape (label) in the test sentence, and *i*-th train sentence, respectively.

#### 5.3.2. Distance of Pdfs Using Komogorov–Smirnov Statistic

Viewing histogram $h_{i}$ as the empirical conditional pdf of the hand shapes, given that the sentence $s_{i}$ has been signed in the SL video, we can use statistical tools such as the Kolmogorov–Smirnov (KS) statistic [49] that quantifies the distance between the underlying distributions. Specifically, the used KS statistic measures the maximum distance between the cumulative distribution functions of two samples, and in our case can be defined as follows:

(4) $$D_{KS}\left(\tilde{s},s_{i}\right)\equiv \max_{n}\left|\sum_{l=1}^{n}{\tilde{f}}_{l}-\sum_{l=1}^{n}f_{l}^{i}\right|.$$

#### 5.3.3. Document Similarity Using tf-idf

The term frequency–inverse document frequency (tf-idf) is a statistic aiming at quantifying the importance of each word (loosely speaking, the amount of information they carry) in the documents of a corpus [50]. The tf-idf reflects the following general idea: the more concentrated the occurrences of a word in the documents of the corpus, the more relevant the word is for identifying the documents in which it appears. Words that appear frequently only in a limited subset of the collection are relevant to the topic of that particular subset (e.g., words such as “car”, “moon”, “fire”, etc.), while words that appear ubiquitously throughout the collection are generally irrelevant to the meaning of the documents (e.g., “the”, “and”, “with”, etc.).

In our case, considering the sentences as “documents” comprising the hand-shape-label “terms”, the tf-idf statistic for a label-sentence pair $\left(l,s_{i}\right)$, can be defined as follows:

(5) $$\text{tf-idf}\left(l,s_{i}\right)=f_{l}^{i}\times \log\left(\frac{\left|S\right|}{\left|s\in S:l\in s\right|}\right),$$

where S denotes the sentence corpus, |·| denotes cardinality, while the term-frequency component $f_{l}^{i}$ is defined in (2). Viewing the test sentence as an unknown “document”, we use the tf-idf statistic as a weighting factor to quantify its relevance to the sentences of the training corpus via the following dissimilarity metric:

(6) $$D_{\text{tf-idf}}\left(\tilde{s},s_{i}\right)\equiv -\sum_{l=1}^{k}\text{tf-idf}\left(l,s_{i}\right){\tilde{f}}_{l}$$

$D_{\text{tf-idf}}\left(\tilde{s},s_{i}\right)$ takes small values (denoting strong similarity) when labels occurring frequently in the test sentence $\tilde{s}$ are also highly relevant to the training sentence $s_{i}$ (namely labels with high tf-idf values).

#### 5.3.4. LSA

Latent semantic analysis (LSA) is a well-known technique used in natural language processing and information retrieval for mapping high-dimensional document representations to a vector space of reduced dimensionality, called the latent semantic space [51]. The aim of LSA is to find a representation so that terms having a common meaning are roughly mapped to the same direction in the latent space. By revealing the semantic relationship between the involved entities, LSA can lead to meaningful association between pairs of documents, even if at a lexical level, they are totally different [52].

In our experiments, we use LSA to compare the sentences in the lower dimensional latent space. To this end, we first obtain the representation of the training corpus in the latent space via the SVD decomposition of the co-occurrence matrix $H=[h_{1},h_{1},\ldots h_{N}]$, with $h_{i}$ denoting the bag-of-words representations defined in (1), where *N* is the number of training sentences, as $H=U\Sigma V^{T}$. Then by setting all but the *m* largest singular values in Σ to 0, we obtain the lower dimensional mapping of the training corpus as

(7) $$H_{m}=U_{m}\;\Sigma_{m}V_{m}^{T},$$

where $U_{m}$, $\Sigma_{m}$, $V_{m}$ are of dimensions k×m, m×m, N×m, respectively. In this mapping, the columns of $\Sigma_{m}V_{m}^{T}$ correspond to the sentence representations in the latent space. To compare the test sentence to the ones in the training dataset, we first obtain its *m*-dimensional latent representation as $\tilde{s}=U_{m}^{T}\;\tilde{h}$, and then calculate the following metric:

(8) $$D_{LSA}\left(\tilde{s},s_{i}\right)\equiv 1-\frac{s_{i}^{T}\;\tilde{s}}{\left|\right|s_{i}\left||\;|\right|\tilde{s}\left|\right|},$$

where $s_{i}$ denotes the *i*-th column of $\Sigma_{m}V_{m}^{T}$.

## 6. Experiments

In this section, we present the experimental evaluation of the proposed dialogue act classification and sentence recognition system on the HealthSign dataset comprising simulated psychiatric interviews for deaf and HoH patients.

### 6.1. Experiment I: Evaluating the Hierarchical Classification of Dialogue Acts

In the first experiment, the classification techniques described in Section 4 were evaluated in terms of accuracy, using the available dataset. Although the dataset consisted of both doctor and patient sentences, as discussed in Section 4, here we focused on doctor sentences. For an unbiased evaluation of the classification accuracy, we considered only the unique sentences in the classification schemes, omitting repetitions that occur among the conversation scripts. Specifically, the dataset comprised 430 unique doctor sentences distributed into 25 classes. Since the training data size was relatively small and each class had a varying nonbalanced number of sentences, we adopted a leave-one-out cross-validation (LOOCV) strategy for our evaluation.

**Figure 3:**
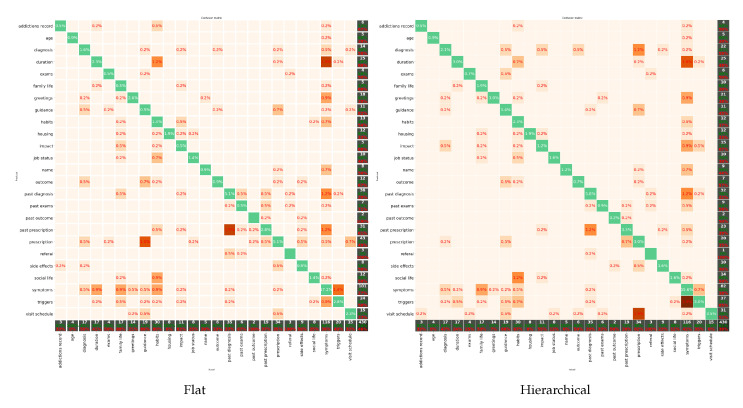
Confusion matrices of the flat (**left**) and hierarchical (**right**) classifiers. Reprinted with permission from [14]. Copyright 2021 Association for Computing Machinery (ACM).

The chance level was estimated at 27% since the class *symptoms*, which was the largest in the dataset, contained 116 out of 430 unique sentences (27% of our dataset). In this sense, both the classification schemes were way above chance level. The flat classification scheme, using a value of k=15 for our *k*-NN classifier, achieved an accuracy of 54.4%, while the accuracy of the hierarchical classification amounted to 60.9%. Thus, the experimental results revealed a performance gap of 6.5% between the hierarchical classification scheme and its flat rival. Deeper analysis into our results also revealed that sentences with similar vocabulary led the classifiers to misclassify them to neighboring classes, as illustrated by the confusion matrices shown in Figure 3.

### 6.2. Experiment II: Evaluating the Context-Aware Sentence Recognition System

The aim of this experiment was to assess the accuracy of the proposed method using a sentence recognition system and to assess the impact of incorporating prior knowledge in our solution. To this end, we used a dataset of 144 annotated SL videos consisting of 8 signers enacting 18 simulated scenarios. For evaluation purposes, we followed a LOOCV strategy, whereby we used the sentences of a particular signer as a testing set and the sentences of the remaining signers as the training set. We repeated this procedure for all eight available signers. The distribution of the training/testing dataset sizes (in number of sentences) is shown in Table 2. As it can be observed, we had in average an 88–12% split of the dataset, with the small variations being attributed to the different way used by each signer to sign the scenario sentences, as well as small discrepancies in the video annotation process. In this setup, as prior knowledge, we considered the true topic labels of the patient sentences as a way to showcase the full potential of such a retrieval system that incorporated a priori information.

### 6.3. Results

**Figure 4:**
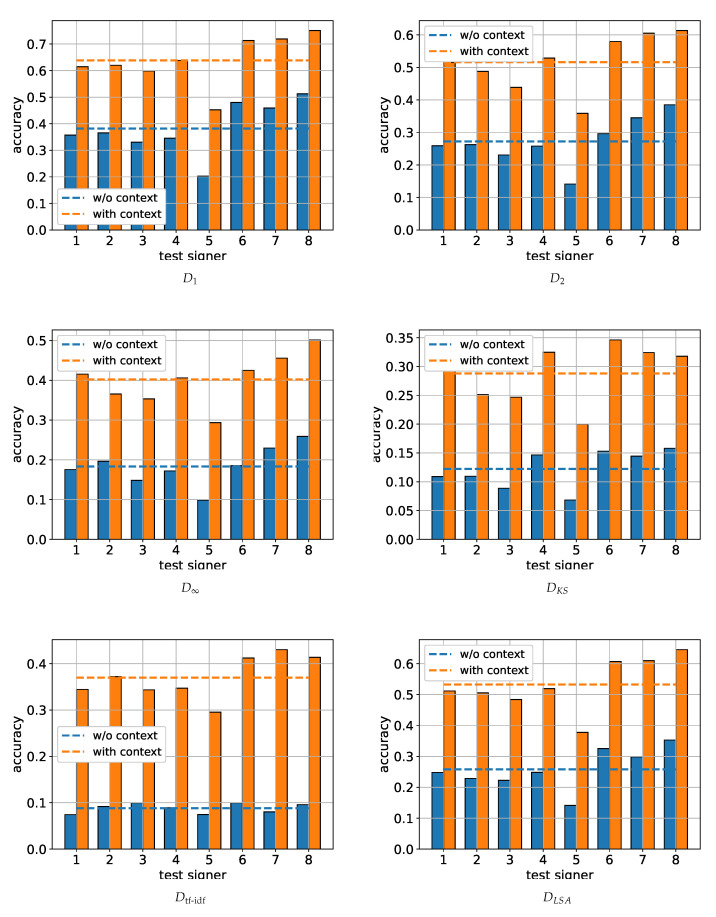
System evaluation via the LOOCV strategy using the six metrics defined in Section 5.3. In all cases, the number of clusters (latent hand shapes) was equal to k=125, while the dimension of the latent space for $D_{LSA}$ was set to m=50.

As mentioned in Section 5, the sentence recognition task was performed by assigning the unknown sentence to its closest neighbor in the training set via the minimization of a preselected dissimilarity metric. When the prior knowledge was used, namely when the unknown sentence had been assigned the dialogue–act class inferred from the preceding doctor query, the search space included only training sentences that belonged to the same class. In our evaluation, we used the six metrics presented in Section 5.3, and experimented with *k* values (i.e., number of clusters/latent hand shapes) in the range [50,150], with the best results being obtained for k=125. Furthermore, the dimension of the latent space for the LSA-based metric was set to m=50 after experimentation. The results obtained for the six used metrics and for k=125 are summarized in Figure 4. As it becomes readily apparent, the use of $D_{1}$ led to the best overall system performance, with a safe margin from the $D_{2}$ and $D_{LSA}$ which was very close in performance at the second place. On the other hand, the $D_{KS}$ and $D_{\text{tf-idf}}$ metrics performed rather poorly in our system.

The fact that the incorporation of the dialogue context in the solution of the sentence retrieval task significantly boosted performance by more than 20% in most cases, regardless of the metric used, underlines the benefits of utilizing prior information when dealing with challenging problems such as the one at hand.

**Figure 5:**
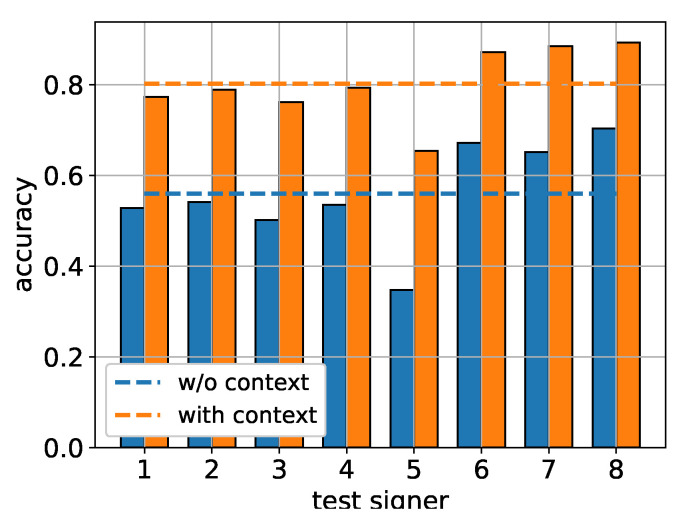
Top-3 accuracy using $D_{1}$ as the distance metric, for k=125.

In absolute terms, the combination of the $D_{1}$ metric with the incorporation of the dialogue context led to an accuracy between 60% and 70% (with the exception of signer five), reaching a peak performance of around 72%. For completeness, in Figure 5 we also present the top three accuracy of the $D_{1}$-based system (i.e., measuring the probability of the test sentence to be correctly identified to one of its three nearest neighbors in the training dataset). As is to be expected, the top-3 results significantly exceed the top-1 accuracy shown in Figure 4, reaching very satisfactory values of around 80% and 90% regarding the mean and peak performance, respectively.

## 7. Discussion

The research presented in this manuscript is part of our ongoing efforts toward SL translation. Given the complexity of the task, a domain-specific approach appears to be meaningful. Based on this principle, we have worked toward an SL translation system to enhance the existing services and help mental health professionals and other clinicians effectively perform a psychiatric evaluation and treat deaf or hard of hearing people.

On a technical level, due to the challenging nature of the problem, purely data-driven methods will require huge amounts of annotated data to capture the basic scenarios. On the other hand, the combination of data-driven information extraction with the encoding and utilization of a priori available knowledge, including linguistic structures as well as domain and context knowledge appears to be promising.

The confinement of psychiatric interviews involving dialogues between (nondeaf) doctors and (deaf) patients offers itself as a domain-specific approach due to its structure based on medical protocols. The domain knowledge by classifying the doctor’s queries in terms of the information they are seeking from the patient, can be captured using a hierarchical ontology. This classification appears to be able to provide useful prior information on the anticipated response from the patient and can be utilized as part of the proposed SL translation system as a sentence retrieval problem.

The system performance becomes significantly better when we incorporate prior knowledge. The challenge appears to be how to capture this knowledge into the system’s knowledge base in more general settings. That requirement may limit its real-world usability, but on the other hand, it seems a feasible alternative to the most difficult data collection and annotation. That alternative becomes attractive, especially when we have to deal with domains entailing structured scenarios.

At a technical level, a source of errors in the proposed pipeline is the feature extraction module since the landmark-based features from hand tracking may not always give correct results in realistic conditions. Another point of concern is that there can be a population imbalance in the classes of the proposed DA classifier, with certain classes being much more present in the corpus than others. In particular, the classes that relate to symptoms, habits, impact, family life are the most frequently occurring in the interview scripts. Other classes, with occurrence in the range [2%, 7%] are past diagnosis/outcomes/exams/prescription, duration, job status, past side effects, addictions record, referral, housing, prescription, while marital status, guidance, childhood, age, greetings, diagnosis, school years, past operation, name, outcome, exams are very rare occupying a small percentage of the total data (lower than 1%).

The presented framework is a work in progress that we are gradually but constantly improving both on the front of the collected data and on the front of its SL translation capabilities. Regarding the data, we are currently in the process of significantly expanding and improving our corpus by including several new scenarios and signers and by enhancing our annotation mechanism. On the technical side, we are working toward an enhanced and refined version of the proposed ontology. Focusing on hierarchical classifier’s confusion matrix (right panel of Figure 3), the classification errors dictate the internal nodes in the hierarchy that could be considered as a unity, simplifying the ontology and enhancing the classification outcome. We observe that there are DAs that share a common path in the graph, and the classification error occurs at the bottom of the hierarchy (leaf nodes). From this observation, we have identified classes in the leaf nodes whose corresponding vocabularies are common and thus could be merged.

Finally, a translation system based on SL recognition is currently under development. The system aims to predict the likelihood of a gloss being present in the patient’s response, given the feature representation of the SL video and the prior PDF of the glosses produced by the DA classification of the doctor’s query. We are also anticipating that the extended dataset will allow for more elaborate schemes for feature extraction such as the use of convolutional neural networks and autoencoders, as well as modeling and fusing additional information streams from the signers’ hand trajectories, and facial expressions that add punctuation information to the feature set.

## 8. Conclusions

In this work, we presented a system for the automatic retrieval of a patient’s response during psychiatric interviews involving deaf and HoH patients. To this end, the general idea behind the proposed system was to infer the dialogue-act class of the doctor’s query, using a hierarchical classification scheme, and then utilize this prior knowledge in order to facilitate the automatic recognition of the patient’s response. The presented system is capable of achieving promising accuracy levels after incorporating the ontological scheme. It appears that this research line has some potential for alleviating the need for more annotated data in low-resource languages such as the SLs.

## Figures and Tables

**Table 1:** An example of annotated interview between doctor (D) and patient (P). The original dialogue is in Greek, and it has been translated by software for illustrative purposes. Reprinted with permission from [14]. Copyright 2021 Association for Computing Machinery (ACM).

Speaker	Dialogue Act	Utterance (Original in Greek)	Utterance (Translation)
D	symptoms	Πώς είναι ο ύπνος σας?	How is your sleep?
P		Τώρα με το χάπι είναι καλός.	Now with the pill it is good.
P		Ξυπνάω ξεκούραστη.	I wake up relaxed.
P		Πριν όμως να πάρω το χάπι, ξυπνούσα πολλές φορές μέσα στη νύχτα.	But before I took the pill, I woke up several times during the night.
D	past diagnosis	Προβλήματα υγείας γνωστά υπάρχουν?	Are there known health problems?
P		Μόνο χοληστερίνη έχω ανεβασμένη.	I only have high cholesterol.
P		Παίρνω φάρμακο.	I take a medicine.
D	past diagnosis	Γνωρίζετε αν συγγενείς σας πρώτου βαθμού είχαν προβλήματα με το άγχος ή με άλλες ψυχικές παθήσεις?	Do you know if your first-degree relatives had problems with stress or other mental illnesses?
P		Μόνο η μητέρα μου ήταν αγχώδης ακριβώς σαν κι εμένα.	Only my mother was anxious just like me.

**Table 2:** Distribution between training and testing datasets in our experiments.

Signer used for testing	1	2	3	4	5	6	7	8
Size of training dataset	4227	4248	4253	4240	4238	4247	4245	4245
Size of testing dataset	622	601	596	609	611	602	604	604

## Data Availability

The data presented in this study are available on request from the corresponding author. The data are not publicly available yet due to technical reasons.
